# Improvement of Mechanical and Acoustic Characteristics of Halloysite Nanotube-Reinforced Polyurethane Elastomer Composites and Their Applications

**DOI:** 10.3390/polym16213025

**Published:** 2024-10-28

**Authors:** Mengchen Ge, Xiaodong Li, Xing Su, Hao Jiang, Yangwei Wang, Fei Han, Meishuai Zou

**Affiliations:** 1 School of Materials Science and Engineering, Beijing Institute of Technology, Beijing 100081, China; gmcv587@hotmail.com (M.G.); 6120220003@bit.edu.cn (X.S.); jiangh@bit.edu.cn (H.J.); wangyangwei@bit.edu.cn (Y.W.); 2 National Key Laboratory of Science and Technology on Material under Shock and Impact, Beijing 100081, China; 3 The Key Laboratory of Biomedical Information Engineering of Ministry of Education, School of Life Science and Technology, Xi’an Jiaotong University, Xi’an 710049, China; 4 Bioinspired Engineering and Biomechanics Center (BEBC), Xi’an Jiaotong University, Xi’an 710049, China

**Keywords:** halloysite nanotubes, polyurethane, microphase separation, composites

## Abstract

Polyurethane incorporated with nanofillers such as carbon nanotubes, basalt fibers, and clay nanoparticles has presented remarkable potential for improving the performance of the polymeric composites. In this study, the halloysite nanofiller-reinforced polyurethane elastomer composites were prepared via the semi-prepolymer method. The impact of different halloysites (halloysite nanotubes and halloysite nanoplates) in polyurethane composites was investigated. Scanning electron microscopy, X-ray diffraction, infrared spectroscopy, electronic universal tensile testing, and acoustic impedance tube testing were employed to characterize the morphology, composition, phase separation, mechanical properties, and sound insulation of the samples. The composite fabricated with 0.5 wt% of halloysite nanotubes introduced during quasi-prepolymer preparation exhibited the highest tensile strength (22.92 ± 0.84 MPa) and elongation at break (576.67 ± 17.99%) among all the prepared samples. Also, the incorporation of 2 wt% halloysite nanotubes into the polyurethane matrix resulted in the most significant overall improvements, particularly in terms of tensile strength (~44%), elongation at break (~40%), and sound insulation (~25%) within the low-frequency range of 50 to 1600 Hz. The attainment of these impressive mechanical and acoustic characteristics could be attributed to the unique lumen structure of the halloysite nanotubes, good dispersion of the halloysites in the polyurethane, and the interfacial bonding between the matrix and halloysite fillers.

## 1. Introduction

Polyurethane elastomer (PUE) has been known as a promising and versatile synthetic polymer in the world [1,2,3,4,5]. It comprises soft segments consisting of polyols and hard segments formed by isocyanates and chain extenders and exhibits microphase separation in its microstructure [6,7,8]. The structure and properties of PUE can be tuned by varying the type and proportion of the soft and hard segments. The extensive utilization of PUE in the applications of coatings [9], bonding agents [10], backing boards or moldings [11], etc. is attributed to its superior viscosity, elasticity, and damping properties. PUE can be utilized as a superior matrix material through the integration of fillers of varying size and shape, including carbon nanotubes [12], microcrystalline cellulose [13,14], and nano-silica [15]. These PUE composites exhibit enhanced properties favored in the fields of rail transit [16], vibration reduction/sound insulation [17], EMI shielding [18], and construction sectors.

In recent years, inorganic filler-reinforced polyurethane has drawn extensive attention, especially the use of desired fillers (low cost, good performance, and high efficiency) instead of high-cost materials as reinforcement. Halloysite nanoparticles have drawn significant research interest as a novel material [19]. Halloysite nanotubes (HNTs) are readily obtainable and abundant and are more affordable than other nanoparticles such as carbon nanotubes (CNTs). Halloysite nanotubes present several advantages over synthetic nanotubes, such as carbon nanotubes (CNTs), in polymer composite applications. First of all, HNTs show superior economic viability due to their low cost (USD 4 HNT vs. USD 500 CNT per kg, reported by Lvov et al. in 2008) [20]. Secondly, HNT and HNT/PU composites are biocompatible and suitable for medical applications, while carbon nanotubes are toxic or harmful to plants, animals, microbes, and human health [21,22,23]. Thirdly, HNTs present excellent availability and make mass-scale industrial application achievable. The global supply of halloysites exceeds a thousand tons per year, while the high-cost CNTs are limited to gram-scale yields [24]. Furthermore, the dimensional size of the HNT tubular porous structure varies from mesoporous (2–50 nm) to macroporous (>50 nm). Compared with other synthetic porous nanomaterials (e.g., CNTs, 2–20 nm), the larger lumen structure of HNTs enables versatile functionality, such as the loading of nanoparticles or immobilization of large enzymes, which could be further utilized in acoustic or medical applications [25,26]. Prior investigations have indicated that the integration of these materials as nanofillers in nanocomposites leads to notable advancements in their mechanical, thermal, and electrical capabilities [27]. As for acoustic properties and performance, scholars have conducted research in this area, and many useful studies were reported [28,29,30,31,32]. Kim and Ahn et al. demonstrated ternary composites of polypropylene 4.8 wt%/clay 0.5 wt% carbon nanotubes with the best soundproofing property among all specimens [33]. The sound transmission loss of the prepared nanocomposites exceeded that of the neat matrix by 15–21 dB in the high-frequency (3200–6400 Hz) and 8–14 dB in the low-frequency (580–620 Hz) range. Liang examined the acoustic insulation characteristics of PVC/glass ball (GB) composites with glass beads and PP/hollow glass ball (HGB) composites with hollow glass beads. The results indicated that the sound attenuation capability of the PP/HGB composite surpassed that of the PVC/GB composite [34]. Yang et al. studied the influence of different HNT contents on the mechanical properties, damping properties, and sound insulation properties of PVC composites [35]. The results showed that when the HNTs content was 8 wt%, it could effectively improve the damping properties and mechanical properties of the composites, and it also exhibited a positive impact on sound insulation performance. These findings proved that hollow fillers (CNT, HGB, HNT) could improve the sound insulation and mechanical properties of the composites. More importantly, the unique crystal structure of HNTs not only resembles that of CNTs in terms of aspect ratio (length to diameter, L/D), but also has a highly ordered structure with a gibbsite octahedral layer of aluminol groups bound in the inner surface and a tetrahedral sheet of silanol groups on the external surface [36]. The moderate density, high aspect ratio, and BET surface of HNT results in higher reinforcing effects and makes it suitable for the preparation of lightweight polymer composites [37,38,39]. Therefore, HNT possesses the potential to serve as an additive enhancing the mechanical properties of polymers and emerges as a promising candidate for a wide variety of potential applications, including sound insulation [40], sound absorption [41,42], molecular adsorption, molecular encapsulation, storage and transportation, as well as serving as a catalyst or supporting reagent in chemical interactions [43].

The impacts of HNT additions to polyurethane in the literature were mainly focused on mechanical studies. The acoustic performance of HNT/PUE composites was reported scarcely, especially the sound insulation properties. A few papers about HNT/PUE composites have been published, but these works are related to sound absorption. In this work, the fabricated HNT/PUE composites not only present competitive mechanical properties but also demonstrate a decent performance in sound insulation and fulfill the research gaps around the sound insulation of HNT-reinforced polymeric composites.

In this study, halloysites with different shapes (halloysite nanotubes, marked as HNT, and halloysite nanoplates, marked as HNP) were introduced to the polyurethane elastomer matrix at various concentrations. The preparation of different halloysites/PUE composites was achieved through the semi-prepolymer process. Rigorous testing demonstrated that all sample properties were effectively improved. Therefore, this work provides a solid foundation for the advancement of vibration damping and noise reduction materials.

## 2. Materials and Methods

### 2.1. Materials

The materials used in this study were sourced from different suppliers. Specifically, methylene diphenyl diisocyanate (MDI), polyether polyol (330 N-4950), and polytetrahydrofuran glycol (PTMG-2000) were obtained from Wanhua Chemical Group Co., Ltd. (Yantai, China). The ultrapure water and 1,4-butanediol (BDO) were acquired from Shanghai Macklin Biochemical Technology Co., Ltd. (Shanghai, China) and applied as a foaming agent and chain extender, respectively. Additionally, the supply of the homogenizing agent silicone oil G-580 was entrusted to Dongguan Guangsiyuan Polyurethane Material Co., Ltd. (Dongguan, China).

The catalyst utilized in this study was a mixture of gel catalyst (T-12) and foaming catalyst (A-1) in a precise proportion. The A-1 catalyst was purchased from Shanghai Zhengui New Material Technology Co., Ltd. (Shanghai, China) and consisted of a 70% solution of double (dimethylaminoethyl) ether and a 30% solution of dipropylene glycol (DPG). The T-12 catalyst was obtained from Xindian Chemical Materials Co., Ltd. (Shanghai, China), and its main component was dibutyltin dilaurate with an effective substance content of 18%. Halloysite fillers (HNT and HNP) were kindly supplied by Mingchuangda Biotechnology Ltd. (Xi’an, China). Although the form of nanotubes are the most common morphology of halloysite. However it has also been reported that there are other various morphologies (spheroidal, platy, etc.) of halloysite from literature [44,45]. The platy halloysites (HNP) were also frequently reported [46,47,48,49]. Moreover, the XRD pattern of the platy halloysites in this study, which was in accordance with literature [50], is attached in Supplementary Figure S1.

It is worth noting that the water and BDO used in this study were analytically pure, while the other chemicals were chemically pure.

### 2.2. Preparation of Polyurethane Composites

The polyols involved in this study underwent vacuum dehydration at a temperature range of 100~110 °C, followed by cooling to ambient temperature, prior to utilization. The polyurethane elastomer matrix was fabricated utilizing the semi-prepolymer method, which involves two stages, prepolymer preparation (stage 1) and a mixing process (stage 2). In stage 1, the dehydration reaction between PTMG-2000 and MDI was conducted under controlled conditions of 80~85 °C with a stoichiometric ratio of R = 6.45, resulting in the formation of the prepolymer designated as component B. In Stage 2, component A, composed of PTMG-2000, 330 N, BDO, ultrapure water, homogenizing agent, and catalyst in a precise proportion, was blended with component B at a ratio of R = 1.05. The mixture was stirred at 2000 r·min^−1^ in a beaker. Then, the homogeneous mixture was poured into a mold, placed at 70 °C for 15 min, and solidified at room temperature for a duration of 48 h.

Polyurethane composite materials consist of a polyurethane elastomer matrix integrated with halloysite particles. HNTs were introduced at different stages (prepolymer preparation, stage 1, or mixing process, stage 2) and with different shapes (T for tubular groups and P for platy groups) and contents (0~2%). Subsequently, 0.5 wt%, 1 wt%, and 2 wt% of HNT (0.48 g, 0.96 g, and 1.94 g) were introduced for each 94.75 g of PUE matrix to prepare different HNT/PUE composites. The studied combinations of HNT and PUE are demonstrated in Table 1.

### 2.3. Scanning Electron Microscope (SEM)

The S-4800 series (field emission scanning electron microscope), manufactured by Mettler Toledo (Greifensee, Switzerland), was utilized for investigation of the surface alterations of the HNT fillers and the cross-sectional morphologies of the composite. Generally, the composite material samples were sliced into small fragments measuring 5 mm × 5 mm in surface area and coated with a thin gold layer. Subsequently, these specimens were placed within a vacuum sample chamber for SEM morphology analysis.

### 2.4. Fourier-Transform Infrared Analysis (FTIR)

Fourier-transform infrared spectroscopy (Nicolet 6700, Thermo Fisher Scientific, Waltham, MA, USA) was applied to analyze the PUE matrix and HNT/PUE composites. The prepared samples were studied in the attenuated total reflection mode with a background of air. The instrument’s resolution was set to 4 cm^−1^, and each sample was measured 32 times over the wavelength range of 450 cm^−1^–4000 cm^−1^.

### 2.5. Mechanical Tests

According to established criterion GBT 528-2009 (identically adopts 1S0 37:2005 and lS0 37:2005/Cor1:2008) [51], which describes the determination method of tensile stress–strain properties for rubber, vulcanized or thermoplastic, the mechanical properties of the analyte were verified by CMT4104 series, the universal testing machine from MTS Industrial Systems Co., Ltd. (Guangdong, China). The average analyte thickness was 2.0 ± 0.2 mm, and the experimental velocity was set to be 500 mm·min^−1^.

### 2.6. Acoustic Performance Tests

In order to study the acoustic performance of the prepared materials, the sound transmission loss (STL) was recorded using a 100 mm diameter acoustic impedance tube (BK4206T, Brüel & Kjær, Nærum, Denmark) for low-frequency detection from 50 to 1600 Hz. The thickness and diameter of the samples prepared were 2 mm and 100 mm, respectively. The STL tests were carried with the following experimental settings: a temperature of 16 °C and a relative humidity of 72%.

## 3. Results and Discussion

### 3.1. Matrix Composition Analysis

**Figure 1:**
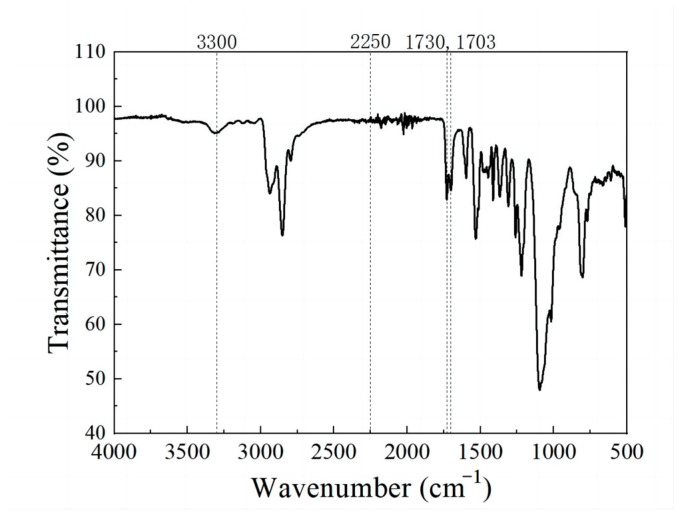
FTIR spectrum of PUE matrix.

The FTIR spectroscopy was conducted on the neat PUE matrix. The resulting spectrum, depicted in Figure 1, revealed the presence of infrared characteristic absorption peaks specific to polyurethane molecules.

In Figure 1, the peak observed at 3300 cm^−1^ corresponds to the stretching vibration of the N-H bond. The peaks at 1730 cm^−1^ and 1703 cm^−1^ are indicative of the presence of ester carbonyl groups [52]. The peak at 1530 cm^−1^ represents the characteristic absorption peak of C-N-H bending vibration. Furthermore, the peak located at 1370 cm^−1^ is ascribed to the symmetrical methyl deformations, and the absence of significant splitting indicates minimal branching in the molecular chains [53]. No obvious absorption peaks occurred at 2250 cm^−1^, which is the characteristic absorption peak of -NCO. The absence of the isocyanate signal indicates that the reaction has proceeded at a high completion. In other words, the designed PUE matrix was successfully synthesized, with minimal monomer residue and side reactions.

### 3.2. Phase Structure Analysis

The phase structure of polyurethane exhibits a close association with the hydrogen bonding force present within the system. In polyurethane, hydrogen bonds are formed by the interaction of amino groups of carbamates, which serve as electron donors, with the strongly electron-withdrawing carbonyl groups serving as electron acceptors [54]. The FTIR spectrum has a high sensitivity to hydrogen bonds, and the detection of hydrogen bonds can indirectly reflect the degree of microphase separation. Therefore, FTIR spectroscopy was used for the analysis of microphase separation in the HNT/PUE composites. This study focuses on two primary spectral regions: N-H absorption and C=O stretching. The amidogen in PUE interacts with the chain extender to form hydrogen bonds, and this interaction mainly occurs in hard segments. This reveals the presence of H-bonding between urethane and urethane or ether oxygen groups. The distinct peak recorded at 3300 cm^−1^ is indicative of the existence of hydrogen-bonded amino groups [55]. It is noteworthy that the spectroscopic analysis revealed no discernible stretching vibration peak corresponding to the free amino group at 3500 cm^−1^ (Figure 1) [56]. This absence suggests that the N-H moiety within the carbamate primarily participates in the formation of hydrogen bonding, since the raw material utilized in this study is polyether polyol rather than polyester polyol. This finding concurs with the conclusions drawn by Clough and Schneider [57].

**Figure 2:**
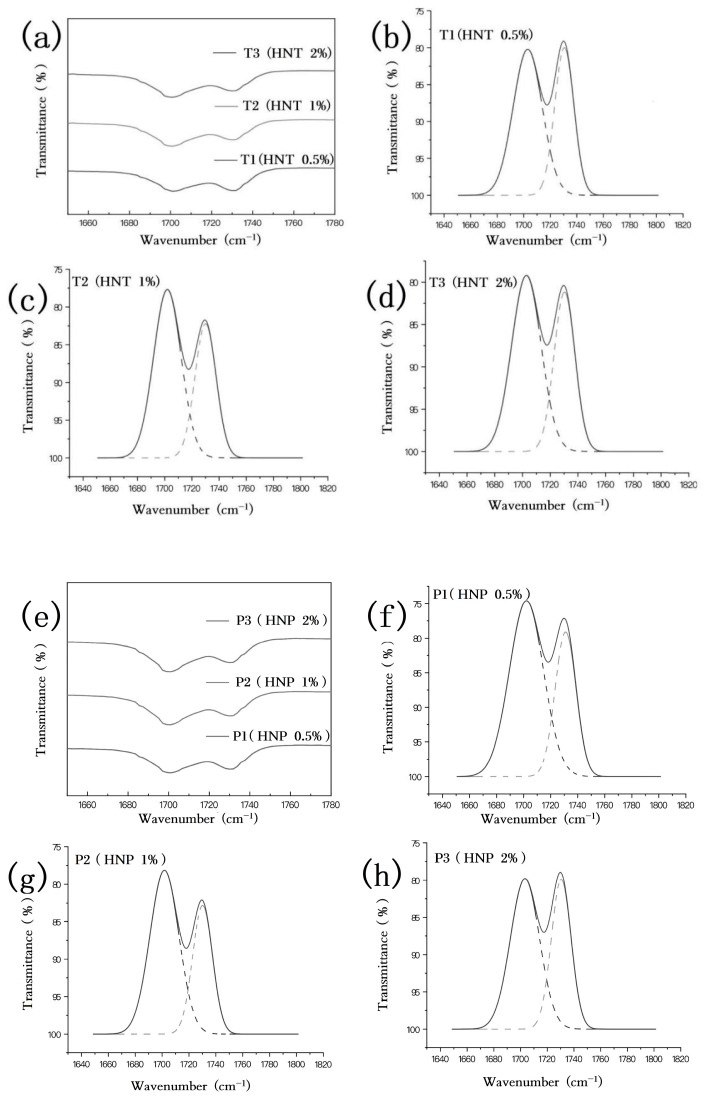
FTIR spectra of the carbonyl part of (**a**) T1–T3; Gauss–Lorentz peak-splitting results of (**b**) T1, (**c**) T2, (**d**) T3; and FTIR spectra of the carbonyl part of (**e**) P1–P3; Gauss–Lorentz peak-splitting results of (**f**) P1, (**g**) P2, (**h**) P3.

In prior studies, some researchers, including Boyarchuk [58], have claimed that polyurethane exhibits a solitary peak for C=O, and all instances are proposed to be hydrogen-bonded. However, Figure 1 displays two distinct C=O peaks. The peak located at 1730 cm^−1^ is attributed to the presence of a free carbonyl group. Furthermore, in cases where the C=O moiety is involved in hydrogen bonding, a second peak is expected to emerge at a slightly lower frequency, approximately 1700 cm^−1^ [59]. To acquire more comprehensive insights into these bands, the Gauss–Lorentz peak-splitting technique was executed using the Origin software. This analysis was focused on the specified wavenumber range of 1780~1650 cm^−1^, which corresponded to the waveband for the carbonyl groups present in the composites, as illustrated in Figure 2a,e. The experimental results are depicted in Figure 2b–d,f–h, with the peak-splitting data being comprehensively outlined in Table 2. The hydrogen-bonding index (HBI) is defined as the ratio of the peak area associated with hydrogen-bonded carbonyl groups to the peak area associated with free carbonyl groups, calculated as HBI = A (bonded C=O)/A (free C=O) [60,61]. The HBI value ought to function as a metric for assessing the extent to which the carbonyl group participates in hydrogen bonding, with higher numerical values indicating a greater involvement of the carbonyl group in hydrogen-bonding interactions.

In Figure 2a,e, the introduction of fillers did not result in any notable alterations in the peak positions. The HBI values of the HNT/PUE composites consistently exceeded those of neat PUE, attributed to the altered hydrogen bonding within the hard segments as a consequence of the incorporation of the HNT fillers. Upon analysis, it is discernible that as the concentration of fillers rose from 0 to 2%, the HBI value initially demonstrated a rising trend, followed by a subsequent decline. The maximum value was achieved when the composition consisted of 1% tubular or 0.5% platy fillers within the PUE matrix. At this point, the HNT/PUE composite exhibited an HBI value of 1.62, with 61.9% of the C=O groups participating in hydrogen bonding. Similarly, the HNP/PUE composite displayed an HBI value of 1.97, indicating that 66.38% of the C=O groups were engaged in hydrogen bonding interactions. The variations in HBI value between the HNT groups and HNP groups is presumably a consequence of their distinct morphological characteristics. Specifically, the HNTs exhibit a folded or curled structure, whereas the HNPs possess an unfolded or layered structure. Consequently, the HNPs possess a greater number of reactive sites, leading to enhanced hydrogen bonding interactions, in comparison with their tubular counterparts. Once the HNT content surpassed that point, the segments were spaced apart at greater distances, leading to a significant reduction in hydrogen bonds. It is noteworthy that as the HBI value increases, greater numbers of hydrogen bonds are involved in the urethane and ether oxygen groups. These hydrogen-bonded hard segments function as effective physical cross-links, thereby restraining the segmental motion within the polymer chain. Consequently, this gives rise to a more pronounced phase separation between the hard and soft segments. A high degree of microphase separation (high HBI values) does not necessarily correlate with superior performance. Instead, moderate phase separation is beneficial to improve the polymer properties.

### 3.3. Sound Insulation Performance and Mechanical Properties

**Figure 3:**
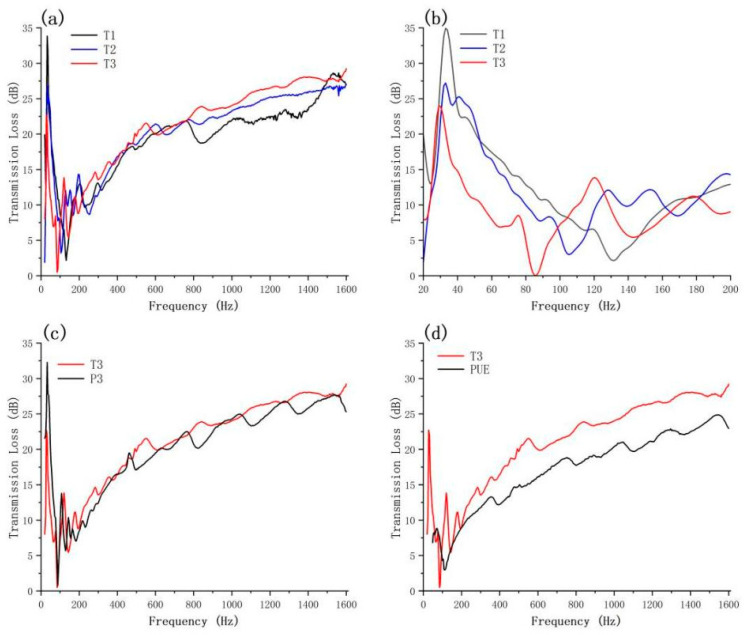
Transmission loss curves (**a**) 0–1600 Hz and (**b**) 28–200 Hz of HNT/PUE with 0.5%, 1%, and 2% HNT content (T1, T2, T3). (**c**,**d**) Transmission loss curves of halloysite/PUE composites (T3 vs. P3 vs. PUE).

The acoustical characteristics of HNT/PUE composites can be effectively represented through STL testing [62]. In this work, the level of sound insulation was measured by employing the standing wave technique. As shown in Figure 3, the materials exhibited a pronounced sound insulation performance across the frequency spectrum ranging from 200 Hz to 1600 Hz. The addition of HNT fillers effectively enhanced the sound insulation capability of the samples. The sound insulation improved as the filler content in the HNT/PUE composites increased. The highest sound insulation was found to be around 30 dB (20% higher than the matrix) at 1600 Hz in 2% HNT/PUE composites. In comparison with the commercial sound-insulation polymers and acoustic-damping materials utilized in aircraft (see Supplementary Figure S3), the HNT/PUE composites prepared in this study, with a density of 1.06 g/cm^3^, exhibit exceptional soundproofing capabilities while being significantly lighter, by 30 to 58%. Specifically, the HNT/PUE composites demonstrate a superior sound reduction performance of approximately 3–5 dB over the commercial sound insulation polymer group, which has a density of 2.48 g/cm^3^, within the frequency range of 330 Hz to 1600 Hz. Additionally, the composites surpass the acoustic damping material applied in aircraft, with a density of 1.51 g/cm^3^, by 5–13 dB within the frequency range of 400 Hz to 1600 Hz.

The variations in overall acoustic performance between the HNT/PUE and HNP/PUE composites are attributed to the different surface morphologies since more surface roughness resulted in more sound reflection. Moreover, the first lowest valley was shifted to a lower frequency as the filler content increased; this indicates that the resonance frequency of HNT/PUE composites is tunable. The incorporation of fillers offers increased opportunities for the reflection of sound waves, thereby extending the propagation distance of sound and ensuring thorough friction of the air within the matrix. The microphase separation exerted a significant influence on the sound absorption performance. The sound wave penetrated the material and underwent numerous reflections within the alternating soft and hard segments. The repeated propagation of the sound wave accelerated the progress of energy consumption. Furthermore, the introduction of fillers improved the microphase separation to a certain degree, and the lumen structure of the HNT enhanced the sound attenuation, benefiting from the air-damping and sound-reflection effects. These resulted in more sound transmission loss, so the sound insulation was improved.

**Figure 4:**
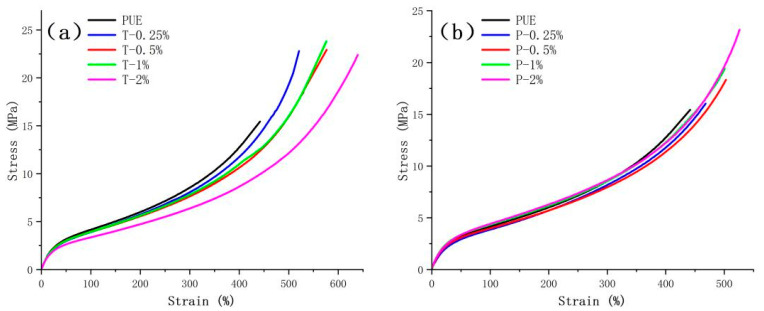
Stress–strain curves of the PUE matrix and different composites with (**a**) HNT and (**b**) HNP.

**Figure 5:**
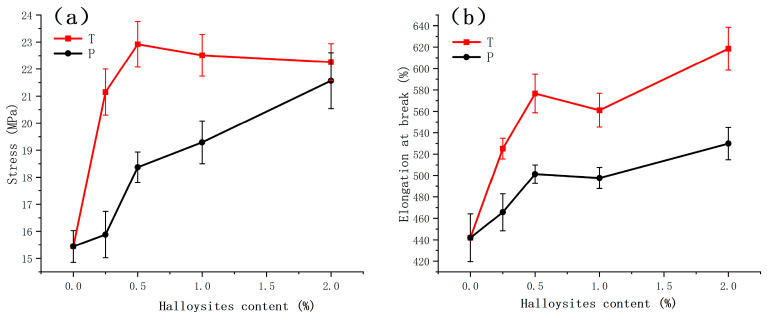
Line graph for comparison of (**a**) tensile strength and (**b**) elongation at break with different filler contents in halloysite/PUE composites.

In actual applications, adequate mechanical properties (i.e., elasticity and toughness) of composite materials are vital [63]. Therefore, a crucial objective in developing HNT/PUE composites is to significantly enhance their mechanical properties with the inclusion of a mere few percent of HNT fillers. For the tensile characteristics of these HNT/PUE composites, a typical stress–strain diagram and a line chart graph of PUE containing different contents of HNT or HNP are shown in Figure 4 and Figure 5, where both the tensile strength and the elongation to break of the different HNT composites increase substantially. Table 3 provides a comparative analysis of the mechanical properties of different PUE composites across various compositions. Furthermore, Figure 4 illustrates the typical rubber-like mechanical behaviors exhibited by these materials, characterized by ductile fracture without any prior yielding. The composites in Figure 4a and Figure 5a were prepared by incorporating tubular HNTs with polyurethane, while the composites in Figure 4b were prepared by incorporating HNP filler. As the filler content increased, the tensile strength of the HNT/PUE composites first increased and then reached a plateau at approximately 22 MPa, while the HNP/PUE groups increased all the time. As for the elongation at break parameter, both HNT/PUE and HNP/PUE composite groups showed the same trend, undergoing an overall increasing trend with a slight decrease at 1% filler content. In contrast with the HNP-reinforced groups, the HNT-reinforced groups exhibit a greater ease in achieving enhancement with minimal filler additions. This is evident from the fact that the 0.5% HNT-reinforced composites demonstrated similar performance, achieving only approximately 3% higher tensile strength compared with the 2% HNT-reinforced composites. The incorporation of HNT fillers improved the overall performance of the composite materials, despite the fact that the smaller size tended to inflict less damage on the polyurethane matrix, whereas the larger size had a higher proclivity for inducing defects.

By incorporating varying quantities (0.25 to 2%) of different halloysite fillers, samples were meticulously prepared, and conclusive results were subsequently derived, as shown in Table 3. The HNT/PUE groups displayed a notable enhancement of tensile strength, from 15.44 to 22.92 MPa, at 0.5% HNT in comparison with neat PUE. And then, the tensile strength started to decrease slightly and reach a plateau at 22.51 MPa at 2% HNT in the composites. Generally, the elongation at break increased almost all the time. When the amount of filler was increased to 1.94 g (T3, 2% HNT content in composites), the improvement in the elongation at break was the most significant, with a value of 618.62%, around a 40.00% enhancement vs. pristine PUE. There are two primary reasons for the alteration of the mechanical properties resulting from the content of fillers. Firstly, it is evident that the aforementioned fluctuating trend observed in tensile strength and elongation at break aligns closely with the findings obtained from the FTIR analysis. Tanaka and coworkers demonstrated a modest amount of data indicating the hydrogen bonds experienced minor slipping under pressure without displaying appreciable hydrogen bond rupture [64,65]. Hence, for fillers present in relatively low concentrations, the enhanced microphase separation is advantageous for attaining superior mechanical properties. Secondly, another aspect primarily concerns the frictional interaction between the fillers and PUE matrix. As the concentration of fillers increases, the friction between them and the matrix becomes predominant, thereby serving to equilibrate the internal stress within the matrix. In the present context, the intricate entanglement of polymer chains poses significant obstacles to their disentanglement, thus enabling a larger quantity of molecular chains to effectively distribute and bear the imposed load. Accordingly, a greater force is required to induce fracture in the material. As the filler content exceeds the threshold value, more aggregates and micropores are generated, resulting in enlarged matrix gaps and defects. These trigger a focused accumulation of stress within the material, subsequently leading to a reduction in its mechanical performance. Therefore, the mechanical properties of composites can be notably enhanced by selecting a suitable filler size and content. Table 4 shows that the composites with HNT introduced at the first stage (prepolymer preparation) have better performance than the composites with HNT introduced at the second stage (mixing stage). This could be attributed to the MDI modification since HNT itself is a good linker and is chemically reactive.

In contrast with other documented filler/polyurethane composites, such as BF/PUE and CNT/PUE, the superior properties exhibited by HNT/PUE, along with the economical cost of the HNT utilized in this study, render it a favorable choice for sound insulation purposes in automotive or architectural domains and presents a viable alternative to CNT filler. Moreover, there is still room for improvement in the mechanical and acoustic performance of this work. The utilization of the lumen structure of HNT can be extended to further enhance the soundproofing capabilities of composites, for instance, through the immobilization of the nanoparticles. Additionally, the implementation of surface modification techniques on HNT, such as silane coupling, contributes to the improvement of the mechanical behavior of these composites.

### 3.4. HNT Surface and Composite Tensile Fracture Interfacial Morphology

**Figure 6:**
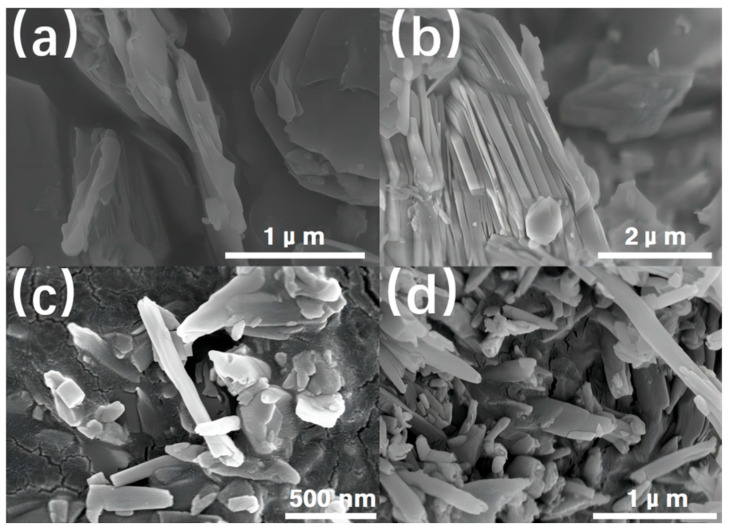
Scanning electron microscope images of (**a**,**b**) HNP and (**c**,**d**) HNT.

**Figure 7:**
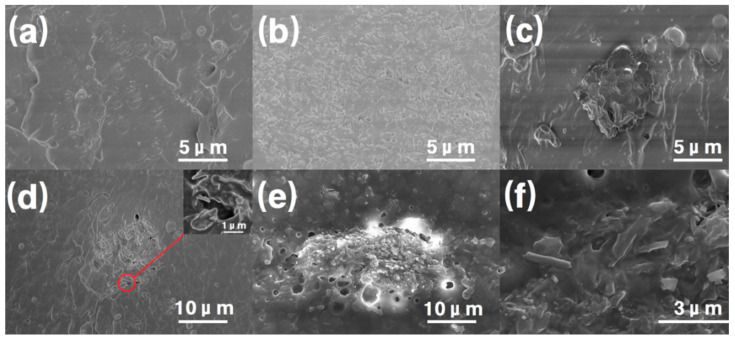
Cross-sectional SEM images of different halloysite/PUE composites: (**a**) T-0.25%, (**b**) T-2%, (**c**) P-0.5%, (**d**) T-0.5%, (**e**) T-0.5%-S2, and (**f**) T-0.5% exhibits a pull-out effect.

The results analysis of the tests conducted revealed that T3, containing 2% HNT, demonstrated the most exceptional overall performance. Therefore, SEM images of the HNT and HNP, as well as the fracture surface of HNT/PUE and HNP/PUE, were taken to compare with those of the matrix, as shown in Figure 6 and Figure 7. According to the particle size distributions graph (Supplementary Figure S2), both HNT and HNP exhibited a distinct bimodal distribution, with one peak in the range of 0.1~10 μm and another peak in the range of 10~100 μm. The different shapes of HNT are presented in Figure 6a–d. It is shown that the HNPs were mainly composed of multilayers of small particles, while the HNTs had a smaller size, with a 100 nm inner diameter and up to around 1 μm length. These findings are in accordance with the distinct bimodal distribution. As for the surface morphology with varied HNT content, the surface of the composites changed from smooth to rough. Furthermore, the appearance and adhesion of fine particles onto the HNP’s surface led to a significant increase in roughness. And some unfolded HNTs were observed as well. This increased the surface area of the fillers as well as the friction between the filler and the matrix. The observed multi-tiered structure of the fillers served as a principal factor contributing to the enhanced performance.

The consistent distribution of the fillers throughout the polymer matrix is crucial for achieving optimal performance in the composite material. Figure 7a–f show the cross-sectional SEM images of the halloysite/PUE (HNT or HNP) composites with different filler contents, exhibiting the characteristics resulting from tensile fracture. Figure 7a,d,b demonstrate the surface morphology of HNT/PUE composites with different filler contents (0.25%, 0.5%, and 2%, respectively). As shown in Figure 7a, there is no obvious agglomeration phenomenon observed in composites with 0.25 wt% addition of HNT, presenting good dispersibility. As the filler content gradually increases, the size of the aggregation area enlarges, and more micropores are generated. This indicates a positive correlation between the amount of HNT added and its degree of aggregation. Figure 7c presents the morphology of 0.5 wt% HNP/PUE composites, which is distinct from the HNT/PUE groups. Compared with Figure 7d,e, it can be seen that the preparation process strongly affects the morphology of the composites. The composites with HNT introduced during stage 2 (mixing process) present obvious agglomeration phenomena and inhomogeneous large micropores, resulting in bad dispersibility in the composites and low performance in their mechanical properties (a decrease of around 43.8% in tensile strength and 22.9% in elongation at break). The composites with HNT introduced during prepolymer preparation demonstrate smaller agglomeration areas and regular morphologies. This could be attributed to the following reasons. Firstly, there is sufficient dispersion in the prepolymer, reducing the agglomeration phenomenon. Secondly, the surface of the HNT also contains trace hydroxyl, and it can further react with the -NCO group to a certain extent during prepolymer preparation. Lastly, there is quite a short time left for complete dispersion if the HNT is introduced during the mixing process, leading to a lower degree of reaction with -NCO and easier agglomeration. Compared with the inset graph in Figure 7d, an obvious pull-out effect is observed in Figure 7f, and the HNT filler that was well wrapped before has been broken into small pieces. During the transmission of sound, the pores in the composites, assisted by the HNTs’ internal cavities, repeatedly reflect sound waves and attenuate them, thereby enhancing the overall sound insulation capabilities. The anisotropic fillers are enclosed within a polyurethane matrix, thereby enhancing frictional properties in all directions and ultimately achieving a balanced distribution of internal stress. It is noteworthy that the fillers exhibit a uniform distribution pattern, devoid of any distinct aggregation tendencies. As a result, the concentration of stress is effectively minimized. Due to the multi-tiered configuration of the HNT fillers, the internal friction within the composite materials increases during stretching, effectively curbing the incidence of pull-out effects.

## 4. Conclusions

This study presented the results and analysis of a series of examinations that explored the individual and combined roles of introduction time, addition amount, internal cavity structure, and morphology differences in the optimization of the acoustic and mechanical performance of polyurethane-based composite materials. The best time to introduce halloysites in the preparation, and the suitable amounts to add, were revealed, and the test results showed that the fabricated composites exhibit a competitive acoustic performance combined with light weight and small thickness. As the filler content increased, the mechanical properties of the HNT-reinforced polyurethane composites were significantly enhanced. These improvements were attributed to the synergistic effects of the interfacial effect, the elasticity of the halloysite/PUE composites, and the microphase-separated structure. Within all combinations of HNT/PUE composites, the overall optimal performance was obtained with the sample with 2% HNT added during prepolymer preparation; the corresponding tensile strength and elongation at break of HNT/PUE composites were increased by 44% and 40%, respectively. Apart from the best mechanical properties, the lightweight HNT/PUE composites showed good acoustic performance, with 2–5 dB sound insulation improvements in the low-frequency range (50–1600 Hz), effectively reducing noise caused by mechanical vibration and other sources. The performance of this composite is promising and is not yet fully realized. In the future, the HNT fillers can be further modified with coupling agents to improve the surface for mechanical enhancement, and the nanoparticles can be utilized for modification of both the surface and inner cavity of the HNTs to boost the acoustic performance. The findings in this study significantly expand the application of HNT-reinforced polyurethane composites and lay the groundwork for the development of advanced acoustic materials based on filler-reinforced polymer materials with lumen structures.

## Figures and Tables

**Table 1:** The composition of each sample prepared.

Sample Name	PUE	T1	T2	T3
		P1	P2	P3
Polyurethane/g	94.75			
Halloysites addition	0	0.48	0.96	1.94
Halloysites content/%	0	0.5	1	2

**Table 2:** Gauss–Lorentz peak-splitting results and HBIs of carbonyl groups.

Sample	Type	Bonded -C=O	Free -C=O	HBI
PUE (0 wt% HNT)	Peak (cm^−1^)	1700	1730	1.37
	Area (%)	57.70	42.10	
T1 (0.5 wt% HNT)	Peak (cm^−1^)	1700	1730	1.49
	Area (%)	59.89	40.11	
T2 (1 wt% HNT)	Peak (cm^−1^)	1700	1730	1.62
	Area (%)	61.9	38.1	
T3 (2 wt% HNT)	Peak (cm^−1^)	1700	1730	1.55
	Area (%)	60.91	39.19	
P1 (0.5 wt% HNT)	Peak (cm^−1^)	1700	1730	1.97
	Area (%)	66.38	33.62	
P2 (1 wt% HNT)	Peak (cm^−1^)	1700	1730	1.85
	Area (%)	64.95	35.05	
P3 (2 wt% HNT)	Peak (cm^−1^)	1700	1730	1.48
	Area (%)	59.61	40.39	

**Table 3:** Mechanical properties of PUE matrix and halloysite/PUE composites.

Sample	Tensile Strength (MPa)	Elongation at Break
PUE matrix	15.44 ± 0.59	441.87 ± 22.26
T-0.25%	21.15 ± 0.85	525.10 ± 9.62
T-0.5%	22.92 ± 0.84	576.67 ± 17.99
T-1%	22.51 ± 0.77	561.05 ± 15.82
T-2%	22.26 ± 0.68	618.62 ± 19.96
P-0.25%	15.88 ± 0.86	465.72 ± 17.19
P-0.5%	18.37 ± 0.56	501.34 ± 8.43
P-1%	19.29 ± 0.79	497.74 ± 9.84
P-2%	21.57 ± 1.03	529.83 ± 15.11

**Table 4:** Mechanical properties of same-content halloysites/PUEs with different preparations.

Sample	P-0.5%	P-0.5%-S2	T-0.5%	T-0.5%-S2
Tensile strength (MPa)	18.37 ± 0.21	14.99 ± 0.73	22.92 ± 0.85	16.52 ± 0.81
Elongation at break (%)	501.34 ± 5.93	435.93 ± 16.53	576.67 ± 17.99	469.19 ± 18.24

S2 indicates that halloysites were introduced at the mixing stage.

## Data Availability

Data are contained within the article.

## Supplementary Materials

The following supporting information can be downloaded at: https://www.mdpi.com/article/10.3390/polym16213025/s1, Figure S1. The XRD pattern of platy halloysites. Figure S2. The particle size distributions for different halloysites fillers. Figure S3. The acoustic performance comparison with commercial polymer sound insulation materials (F10 series from Soundbox Ltd.) and acoustic damping material for aircraft (Provided from Shanxi Aircraft Industry (Group) Co., Ltd. China).
