# Supplementary material for: Improvement of Mechanical and Acoustic Characteristics of Halloysite Nanotube-Reinforced Polyurethane Elastomer Composites and Their Applications
Source: Polymers (Basel). 2024 Oct 28;16(21):3025. doi: 10.3390/polym16213025 (PMC11548139; doi:10.3390/polym16213025)
Supplement: Supplementary file 1 [file polymers-16-03025-s001.zip › polymers-2978186-supplementary.pdf]

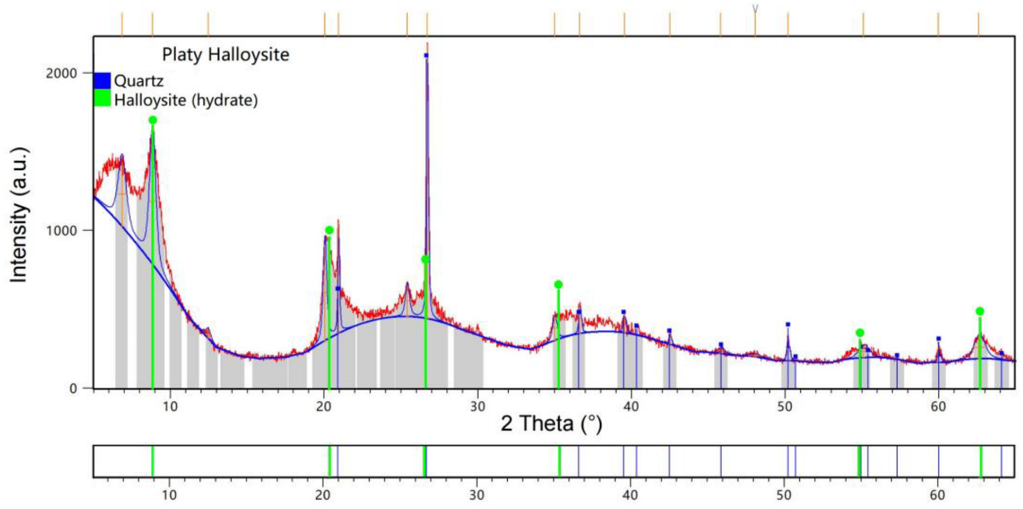

Figure S1. The XRD pattern of platy halloysites

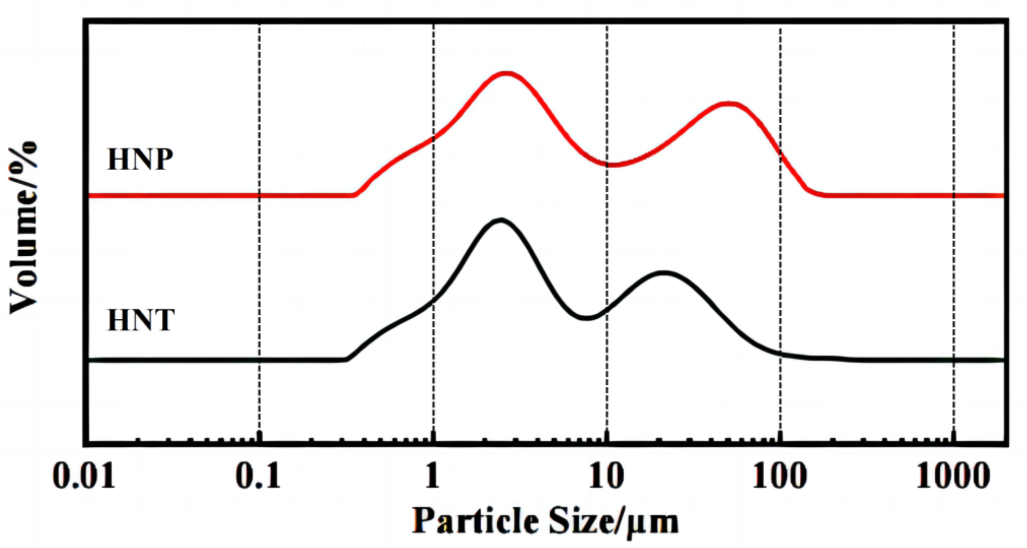

Figure S2. The particle size distributions for different halloysites fillers

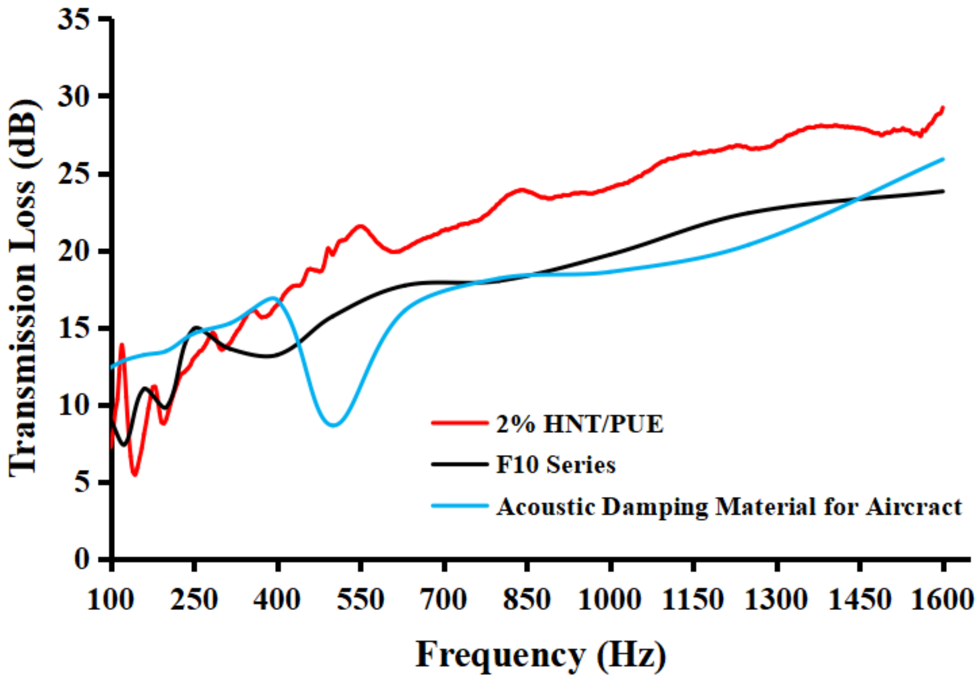

Figure S3. The acoustic performance comparison with commercial polymer sound insulation materials (F10 series from Soundbox Ltd.) and acoustic damping material for aircraft (Provided from Shanxi Aircraft Industry (Group) Co., Ltd China)
